# Loss of Serglycin Promotes Primary Tumor Growth and Vessel Functionality in the RIP1-Tag2 Mouse Model for Spontaneous Insulinoma Formation

**DOI:** 10.1371/journal.pone.0126688

**Published:** 2015-05-15

**Authors:** Andrew Hamilton, Vladimir Basic, Sandra Andersson, Magnus Abrink, Maria Ringvall

**Affiliations:** 1 Department of Medical Biochemistry and Microbiology, Uppsala University, Uppsala, Sweden; 2 Department of Biomedical Sciences and Veterinary Public Health, Swedish University for Agricultural Sciences, Uppsala, Sweden; University of Patras, GREECE

## Abstract

The serglycin proteoglycan is mainly expressed by hematopoietic cells where the major function is to retain the content of storage granules and vesicles. In recent years, expression of serglycin has also been found in different forms of human malignancies and a high serglycin expression level has been correlated with a more migratory and invasive phenotype in the case of breast cancer and nasopharyngeal carcinoma. Serglycin has also been implicated in the development of the tumor vasculature in multiple myeloma and hepatocellular carcinoma where reduced expression of serglycin was correlated with a less extensive vasculature. To further investigate the contribution of serglycin to tumor development, we have used the immunocompetent RIP1-Tag2 mouse model of spontaneous insulinoma formation crossed into serglycin deficient mice. For the first time we show that serglycin-deficiency affects orthotopic primary tumor growth and tumor vascular functionality of late stage carcinomas. RIP1-Tag2 mice that lack serglycin develop larger tumors with a higher proliferative activity but unaltered apoptosis compared to normal RIP1-Tag2 mice. The absence of serglycin also enhances the tumor vessel functionality, which is better perfused than in tumors from serglycin wild type mice. The presence of the pro-angiogenic modulators vascular endothelial growth factor and hepatocyte growth factor were decreased in the serglycin deficient mice which suggests a less pro-angiogenic environment in the tumors of these animals. Taken together, we conclude that serglycin affects multiple aspects of spontaneous tumor formation, which strengthens the theory that serglycin acts as an important mediator in the formation and progression of tumors.

## Introduction

Serglycin is a proteoglycan that is mainly found in storage granules and secretory vesicles of hematopoietic cells, where its principal function is considered to be in the storage of a number of proteases and cytokines [1]. Its expression has also been found in other cell types including human endothelial cells [2] and quite recently in several types of carcinoma [3–5].

The 17 kDa core protein of serglycin has a number of glycosaminoglycan (GAG) chains attached via a serine/glycin rich domain [6]. The predominant GAG type on serglycin is chondroitin sulfate, although other GAGs including heparan sulfate and heparin can also be present depending upon the cell type that expresses it [7]. Mice deficient in serglycin are fertile and viable with no apparent pathologies or behavioral alterations. At the cellular level, the main distinctive phenotype is a loss of storage and/or altered release of compounds such as proteases—elastase in neutrophils [8], granzyme B in cytotoxic T-cells [9] and several mast cell specific proteases [10]. In addition, loss of serglycin gives decreased platelet factor 4 levels in platelets [11], and macrophages have an altered release of tumor necrosis factor-α (TNF-α) upon stimulation with lipopolysacharide (LPS) [12]. Unexpectedly, these changes do not seem to have any severe impact on the functionality of the immune system when challenged with infections from various pathogens [8, 11, 13]. Although serglycin is generally regarded as a committed intracellular proteoglycan, it has additionally been discovered to locate to the surface on different types of tumor cells, both *in situ* [4] and in culture [4, 14].

It has recently been shown that a high expression of serglycin induces a more aggressive behavior of human breast [4] and nasopharyngeal carcinoma (NPC) [5] cell lines, and that NPC liver metastases have a higher expression of serglycin than NPC primary tumors suggesting that increased serglycin expression may contribute to an enhanced invasive phenotype. Additionally, in a clinical study of patients with hepatocellular carcinoma (HCC), high expression of serglycin correlates with a poor prognosis [3]. The mechanism by which serglycin alters tumor cell aggressiveness is still elusive, however differential expression of serglycin seems to affect the expression levels of markers of epithelial to mesenchymal transition both *in vitro* and *in vivo* [3–5].

An important aspect of tumor growth is the ability of the tumor to induce vascular development in order to receive a supply of oxygen and nutrients. Physiological angiogenesis, such as during the menstrual cycle and wound healing, is tightly regulated by different well-balanced pro- and anti-angiogenic factors. However, in pathological situations such as tumor growth, this balance is disturbed and a microenvironment with a high pro-angiogenic pressure is created, which induces the formation of a distorted and hemorrhagic vasculature [15]. Serglycin has been implicated in tumor vessel expansion in addition to its effect on tumor cell aggressiveness. Purushothaman *et al*. [16] have presented an *in vivo* study where human multiple myeloma cells were allowed to form subcutaneous tumors in a mouse xenotransplant model. Tumors formed by these myeloma cells where the expression of serglycin had been knocked down grew smaller and had a less expanded vasculature than the tumors derived from the parental cell line with a higher serglycin expression. In addition, in the clinical study on the effects of serglycin expression levels in HCC, He *et al*. saw that tumors with a less pronounced serglycin expression level were also less vascularized [3]. Taken together, these studies point towards a mechanism whereby serglycin may modulate the tumor vasculature and thereby alter tumor cell behavior and tumor growth.

The RIP1-Tag2 mouse [17] is a well characterized model of pancreatic insulinoma in which tumors form spontaneously and orthotopically. It is therefore considered to be a very appropriate *in vivo* model for multi-step carcinogenesis and as such this mouse strain has frequently been used for studies of the induction and progression of tumor angiogenesis. Transgenic expression of the oncogenic SV40 T antigen (Tag) under the control of the rat insulin promoter leads to a specific expression of Tag in the beta cells of the islets of Langerhans eventually causing development of malignant carcinomas. From 4–5 weeks after birth, the beta cells in all islets (~400) start to hyper-proliferate and a fraction (~10%) of these hyperplastic islets take on an angiogenic phenotype (the angiogenic switch). A number of these angiogenic islets then further progress into tumors. From around 12 weeks of age, all stages of tumor development are seen, from hyperplastic islets, to angiogenic islets, benign encapsulated tumors and invasive carcinomas.

In the present study, we have addressed how serglycin affects spontaneous tumor progression in an immunocompetent mouse model. We have analyzed late stage tumors in wild type and serglycin deficient mice in the RIP1-Tag2 model at a time point where invasive carcinomas have developed. Our results show that the presence of serglycin suppresses spontaneous tumor growth and affects angiogenesis and vascular function *in vivo*. RIP1-Tag2 positive mice that are serglycin deficient (RT2^pos^SG^ko^) develop a larger tumor mass with a better perfused vasculature than wild-type RIP1-Tag2 positive (RT2^pos^SG^wt^) mice. The tumors from serglycin deficient mice had decreased levels of vascular endothelial growth factor A (VEGF) and hepatocyte growth factor (HGF), which suggests a less pro-angiogenic environment. We also observed a lower frequency of angiogenic islets in pancreas from RT2^pos^SG^ko^ than in RT2^pos^SG^wt^ mice, which also is indicative of a lower pro-angiogenic pressure. Our data for the first time suggest that the presence of serglycin can suppress tumor expansion, and is of importance for the functionality of the tumor vasculature.

## Materials and Methods

### Mice

To generate serglycin deficient mice with potential for spontaneous tumor development, we crossed RIP1-Tag2 positive (RT^pos^) male mice with females from a serglycin-deficient knockout mouse strain [10]. All females that were used for breeding were RIP1-Tag2 negative since gestation is too stressful for tumor bearing females. RT ^pos^SG^+/-^ males were further mated with RT^neg^SG^+/-^ females to produce RT^pos^SG^wt^ and RT^pos^SG^ko^ mice. All mice were on a pure C57BL/6 genetic background and littermates were used as far as possible for the analyses. All data presented in this study are derived from 15 week old male mice, a time point at which all of the different stages of tumor formation are present, from hyper-proliferative islets to large carcinomas. From 10 weeks of age and onwards, all RIP1-Tag2 positive mice received drinking water supplied with 5% sucrose to relieve hypoglycemia induced by the insulin-producing tumors, as is common practice with this model. Animals were under daily ocular inspection of their general health, and no differences were observed in the health between the two genotypes. DNA extracted from tail biopsies was used as template for genotyping by PCR. The following primers were used: SG -797 F 5'-gtctctgttttcacattccacggccc-3', SG -482 R 5'-ggcacaagcagggaacattccgagc-3', SG Neo R 5'-gggccagctcattcctcccactcatgatct -3'; Tag2 F 5'-ggacaaccacaactagaatgcag-3', Tag2 R 5'-cagagcagaattgtggagtgg-3'.

### Ethical Statement

All animals were handled in strict accordance with good animal practice as defined by the relevant national and local animal welfare bodies. All animal work was approved by the Uppsala University board of animal experimentation (C 319/10, C 57/13 and C 56/14) and thus performed according to the United Kingdom Coordinating Committee on Cancer Research (UKCCCR) guidelines for the welfare of animals in experimental neoplasia [18].

### Antibodies

Primary antibodies: Rabbit polyclonal anti- Ki67, final dilution 1:500 (ab15580, Abcam. Immunogen: synthetic peptide conjugated to Keyhole Limpet Haemocyanin (KLH) derived from within residues 1200–1300 of Human Ki67 (mouse cross-reactivity)); Rabbit polyclonal anti cleaved caspase-3, final dilution 1:500 (ab13847, Abcam. Immunogen: synthetic peptide corresponding to Human active + pro Caspase 3 residues 150–250 conjugated to KLH (mouse cross-reactivity)); Rat monoclonal anti-CD31, final dilution 1:1000 (557355, BD Pharmingen. Immunogen: 129/Sv mouse-derived endothelioma cell line tEnd.1); Rat monoclonal anti-F4/80, final dilution 1:1000 (MCA497, AbD Serotec. Imunogen: Thioglycollate stimulated peritoneal macrophages from C57BL/6 mice); Rat monoclonal anti-Gr1, final dilution 1:1000 (553123, BD Pharmingen. Immunogen not reported); Rabbit polyclonal anti-VEGF-A, final dilution 1:1000 (sc-512, Santa Cruz Biotechnology. Immunogen: epitope mapping at the N-terminus of human VEGF-A (mouse cross-reactivity)); Rabbit polyclonal anti-HGF, final dilution 1:1000 (ab83760, Abcam. Immunogen: Synthetic peptide corresponding to a region within the N-terminal sequence 108–157 of human HGF (mouse cross-reactivity)); Rabbit polyclonal anti-β-actin, final dilution 1:5000 (ab8227, Abcam. Immunogen: Synthetic peptide derived from within residues 1–100 of human β-actin (mouse cross-reactivity)). Secondary antibodies: goat anti-rabbit Alexa Fluor 488, final dilution 1:500 (A11008, Invitrogen); goat anti-rabbit Alexa Fluor 594, final dilution 1:500 (A11012, Invitrogen); goat anti-rat Alexa Fluor 488, final dilution 1:500 (A11006, Invitrogen); goat anti-rat Alexa Fluor 594, final dilution 1:500 (A11007, Invitrogen); Donkey anti-goat IRDye 680RD, final dilution 1:10 000 (926–68074, LI-COR); Donkey anti-rabbit IRDye 800CW, final dilution 1:10 000 (926–32213, LI-COR).

### Dissection and quantification of angiogenic islets and tumors

Mice were anesthetized by intraperitoneal injection with 2% avertin. Heart perfusion was performed with 10 ml of phosphate buffered saline (PBS; pH 7.4) followed by 10 ml of 2% paraformaldehyde (PFA) in PBS (pH 7.4) and the pancreas was excised. Pancreata were dissected under a stereo dissection microscope at 10× magnification, and the number of tumors (≥1 mm diameter) and angiogenic islets (defined as blood containing hyperplastic isles with a diameter of <1 mm) were counted. Tumors were counted and measured and volumes were calculated using the formula *(π/6)×width* ^*2*^ *×length*. All tissues were stored in a 30% sucrose in PBS solution over night at 4°C. The material was frozen in OCT Cryomount (00890, Histolab) the following day and stored at -80°C until further processed.

### Cell culture

Beta-TC-6 cells were cultured in DMEM media (Invitrogen) supplemented with 15% fetal calf serum (Saveen Werner), 5mM GlutaMAX (Invitrogen) and 5% penicillin/streptomycin (Invitrogen) at 37°C, 5% CO_2_.

### Total RNA extraction and cDNA synthesis

Specimens obtained from pancreatic insulinoma and spleen tissue (2-5mg) were cut in pieces and manually disrupted using a pestle. Total RNA was isolated from the homogenized tumor material and from cultured beta-TC-6 cells using an Omega E.Z.N.A total RNA extraction kit (Omega Bio-Tek) according to the manufacturer’s instructions. The total RNA was quantified using Nanodrop spectrophotometer (Thermo Fisher Scientific). Isolated total RNA was stored at -80°C until further use. 400ng of total RNA was reverse-transcribed using iScript cDNA synthesis kit (BioRad) according to the manufacturer’s instructions. Synthesized complementary DNA was stored at -20°C until further use.

### qPCR analysis

Analysis was performed on ABI Prism Sequence Detection System 7900HT (Life Technologies). Pre-mixed primers and probes set targeting murine serglycin transcript (Srgn) (catalogue number Mm01169070_m1) was provided as Assay-on-demand (Life Technologies). The probes were labeled using FAM as the reporter dye and MGB as the quencher dye. Gene expression analysis was normalized to the expression levels of ribosomal protein large P0 (Arbp/Rplp0—catalogue number Mm00725448_s1). Arbp was chosen as the optimal housekeeping gene from a panel of common endogenous control genes (TATA Biocenter). Each sample was analyzed in duplicate under the following conditions: Initial denaturation for 20s at 95°C, followed by 3s at 95°C and 30s at 60°C for forty cycles. Reactions were performed in MicroAmp optical 384-well reaction plates (Life Technologies).

### Immunofluorescent staining

5μm frozen sections of tumor tissue were fixed with 100% ice cold methanol for 10 minutes, then blocked in 5% normal goat serum in PBS (pH 7.4) for 1h. Sections were incubated with primary antibody at 4°C overnight. Omission of the primary antibody acted as a negative control in all experiments. Sections were incubated with primary antibodies overnight at 4°C and for 1h with secondary antibodies at room temperature. Incubation with 2μg/ml Hoechst in PBS (pH 7.4) for 30 minutes was used to visualize nuclei. Washing was performed in PBS (pH 7.4) between all steps.

### Quantification of proliferation and apoptosis

The proliferative and apoptotic activity in tumor tissue was assessed by counting the number of Ki67 and cleaved caspase-3 positive cells respectively. The data is presented as number of cells/mm^2^. Counting was automated using ImageJ software (National Institute of Health). Four different fields from four tissue levels in each specimen were counted.

### Quantification of vascular perfusion

Mice were anesthetized by intraperitoneal injection with 2% avertin. 100μl of 1mg/ml fluorescein isothiocyanate (FITC)-conjugated tomato lectin (*Lycopersicon Esculentum*; L0401, Sigma) was administrated by retro-orbital injection and allowed to circulate for 2 minutes. Heart perfusion was performed with 10ml PBS (pH 7.4) followed by 10ml of 2% PFA in PBS (pH 7.4) and the whole pancreas containing tumors was excised and frozen as described above. 5μm frozen tissue-sections were stained with an anti-CD31 antibody as described above and the areas of CD31- and FITC-positive staining were quantified using ImageJ (NIH).

### Western Blotting

Excised tumor tissue was homogenized in ice-cold lysis buffer (Sigma Aldrich) containing a protease inhibitor cocktail (P2714, Sigma Aldrich) and centrifuged at 10 000g for 10 minutes at 4°C. Supernatant was separated from the pellet and used in further steps. Total protein concentration was determined using BCA protein assay (Thermo Fisher Scientific). Equal amounts of total protein (40–60μg) were separated under reducing conditions using 12% SDS page and transferred onto Immobilon PVDF membrane (Millipore) in a transblot electrophoretic transfer cell (Bio-Rad Laboratories). Membranes were probed overnight at 4°C with antibodies directed against VEGF-A and HGF with β-actin used as a loading control. Donkey anti-rabbit secondary 800 CW and donkey anti-goat secondary 680 RD, were applied for 60 minutes at room temperature to visualize the signal from the primary antibodies. Visualization and quantification was performed using an Odyssey H scanner and Image Studio Lite software (LI-COR).

### Statistical analyses

GraphPad Prism was used to perform the non-parametric Mann-Whitney test on all data with the exception of the statistical analysis of number of mice with no angiogenic islets, where Fisher's exact test was used. Data are presented as mean±SEM.

## Results

### Generation of serglycin-deficient RIP1-Tag2 mice

To investigate how a systemic lack of serglycin affects different parameters important for tumor progression in an *in vivo* mouse-model system for spontaneous tumor development, we crossed female serglycin knockout mice with males from the transgenic RIP1-Tag2 strain. After sequential breeding, we generated RIP1-Tag2 positive mice that were homozygous for either the serglycin wild-type (RT2^pos^SG^wt^) or knockout alleles (RT2^pos^SG^ko^), both genotypes with a capacity to develop tumors. We found that lack of serglycin did not affect the penetrance for tumor development, which was 100% in both groups.

### Enhanced tumor growth and proliferative activity in serglycin deficient RIP1-Tag2 mice

**Fig 1:**
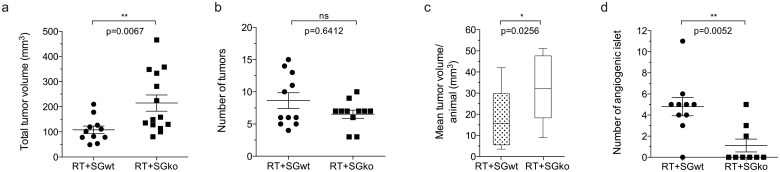
Tumor burden and number of angiogenic islets. Loss of serglycin increases total and mean tumor volume, and decreases the number of angiogenic islets in RIP1-Tag2 mice. Tumors and angiogenic islets were excised from RT^pos^SG^wt^ and RT^pos^SG^ko^ mice at 15 weeks of age and measured. Individual tumor volumes were calculated using the formula *(π/6)×width* ^*2*^ *×length*, which was then used to determine total tumor volume (a). The frequency tumors (b) was also measured and used to calculate mean tumor volume per mouse (c). The number of angiogenic islets was also counted (d). Each data point in represents an individual animal. Statistical analysis was performed using a two-tailed Mann-Whitney test. Error bars represent mean ± SEM.

**Fig 2:**
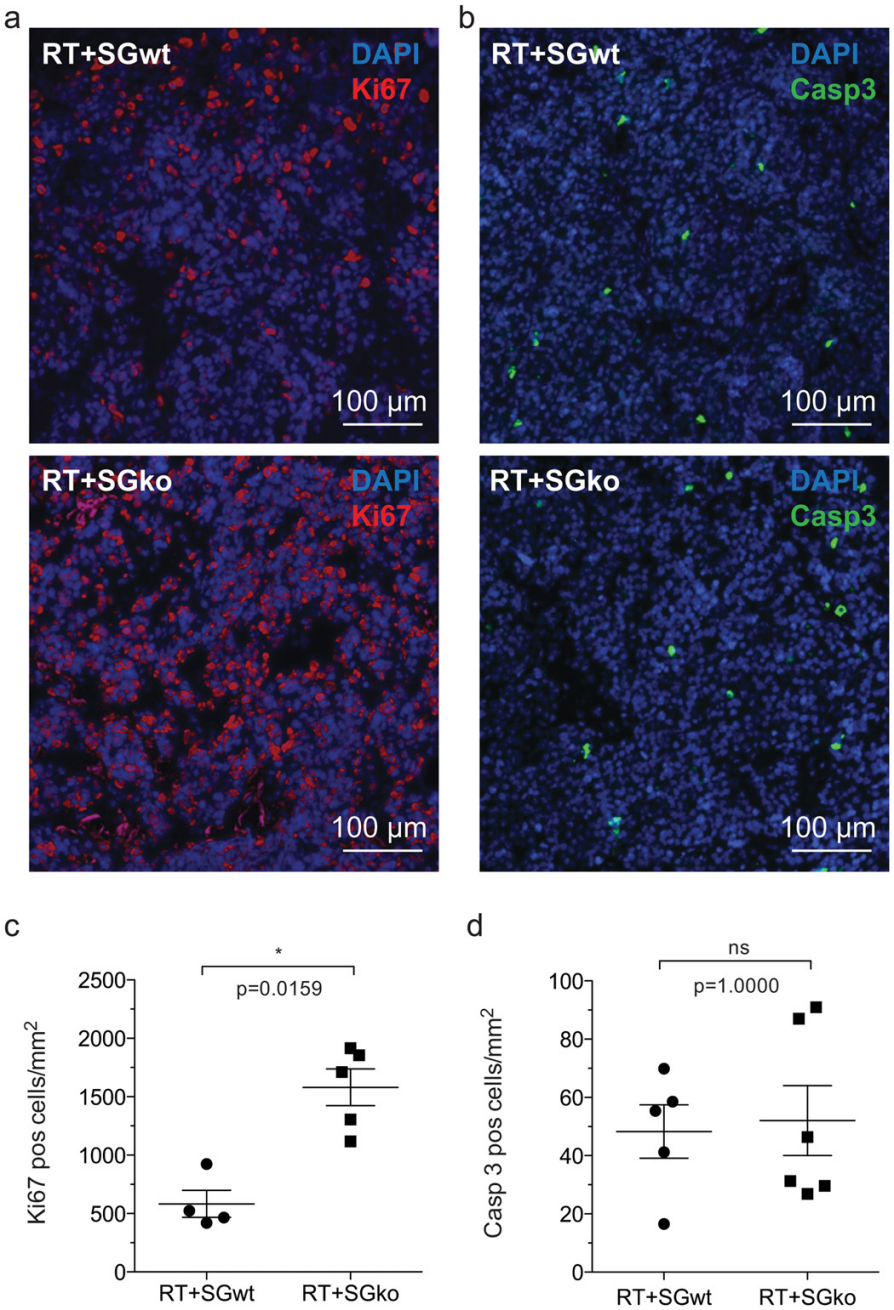
Proliferative and apoptotic status of tumor tissue. The proliferative activity is increased in tumors from RT^pos^SG^ko^ mice compared to those from RT^pos^SG^wt^ mice. Tumor sections from RT^pos^SG^wt^ and RT^pos^SG^ko^ mice were stained for apoptotic and proliferative activity using antibodies against Ki67 (a) and cleaved caspase-3 (Casp-3, b) respectively. Positive cells were counted and tumors from serglycin deficient animals had a significantly increased levels of proliferation (c) while no difference was detected in apoptosis between the two genotypes. Statistical analysis was performed using a two-tailed Mann-Whitney test. Error bars represent mean ± SEM.

Multiple β-cell tumors are formed in the pancreas of RIP1-Tag2 mice and the tumor burden of each mouse can be assessed by calculation of the number and total volume of tumors. To detect any possible differences in tumor burden between the genotypes we dissected pancreata of 15 weeks old mice, a late time point when all stages of lesions are generally present. All tumors were dissected out from each animal in the RT2^pos^SG^wt^ and RT2^pos^SG^ko^ groups respectively. The frequency and volume of tumors was measured to give the total tumor volume per individual. We found that the total mean volume in the RT2^pos^SG^ko^ group was twice that of the RT2^pos^SG^wt^ group (Fig 1a) while the number of tumors per animal did not differ (Fig 1b). The increased tumor volume seen in RT2^pos^SG^ko^ mice is therefore caused by an elevated growth of separate tumors rather than a higher total number of tumors in each RT2^pos^SG^ko^ animal, consequently giving an increased mean tumor volume in RT2^pos^SG^ko^ (Fig 1c). To determine whether this was due to enhanced proliferation and/or decreased apoptosis in the tumors in the RT2^pos^SG^ko^ mice, we performed immunostaining of tumor tissue sections against the proliferation marker Ki67 and the apoptosis marker cleaved caspase-3 (Casp3). While we could see a significantly higher number of Ki67 positive cells/mm^2^ (Fig 2a and 2c) in RT2^pos^SG^ko^ than in RT2^pos^SG^wt^ tumors, we observed no difference in number of Casp3 positive cells (Fig 2b and 2d) between the groups. We therefore concluded that it was enhanced proliferation and not decreased apoptosis that caused the enlarged tumor volume seen in RT2^pos^SG^ko^ mice.

### The frequency of angiogenic islets is decreased in serglycin deficient RIP1-Tag2 mice

Increased angiogenesis is required in order to supply a proliferative cell mass such as a tumor with the oxygen and nutrients it needs for growth. The angiogenic switch, when a hyperproliferative mass is transformed into an angiogenic phenotype, is therefore seen as a determinant for further tumor progression. The frequency of angiogenic islets (i.e. the number of hyperplastic islets that have passed through the angiogenic switch) can be used as a measurement of the overall angiogenic pressure and therefore in addition to counting the number of tumors, we also measured the frequency of angiogenic islets in 15 week old mice. We detected fewer angiogenic islets in the RT2^pos^SG^ko^ than in the RT2^pos^SG^wt^ group, a difference apparent from both comparison of the mean number of islets in the two groups (Fig 1d) and by the fraction of mice where no angiogenic islets were detected (Fisher’s exact test; 8% in RT2^pos^SG^wt^ mice versus 54% in RT2^pos^SG^ko^ mice, p = 0.0272). The lower number of angiogenic islets found in the serglycin deficient animals suggests that there is a less pro-angiogenic environment in these mice as a lower angiogenic pressure causes fewer hyperplastic islets to transform into an angiogenic phenotype. Taken together, the lower number of angiogenic islets and the unaltered number of tumors in the RT2^pos^SG^ko^ mice showed that the total number of lesions on average was significantly lower in the RT2^pos^SG^ko^ group (S1 Fig).

### Tumor vessel functionality is enhanced in serglycin deficient RIP1-Tag2 mice

**Fig 3:**
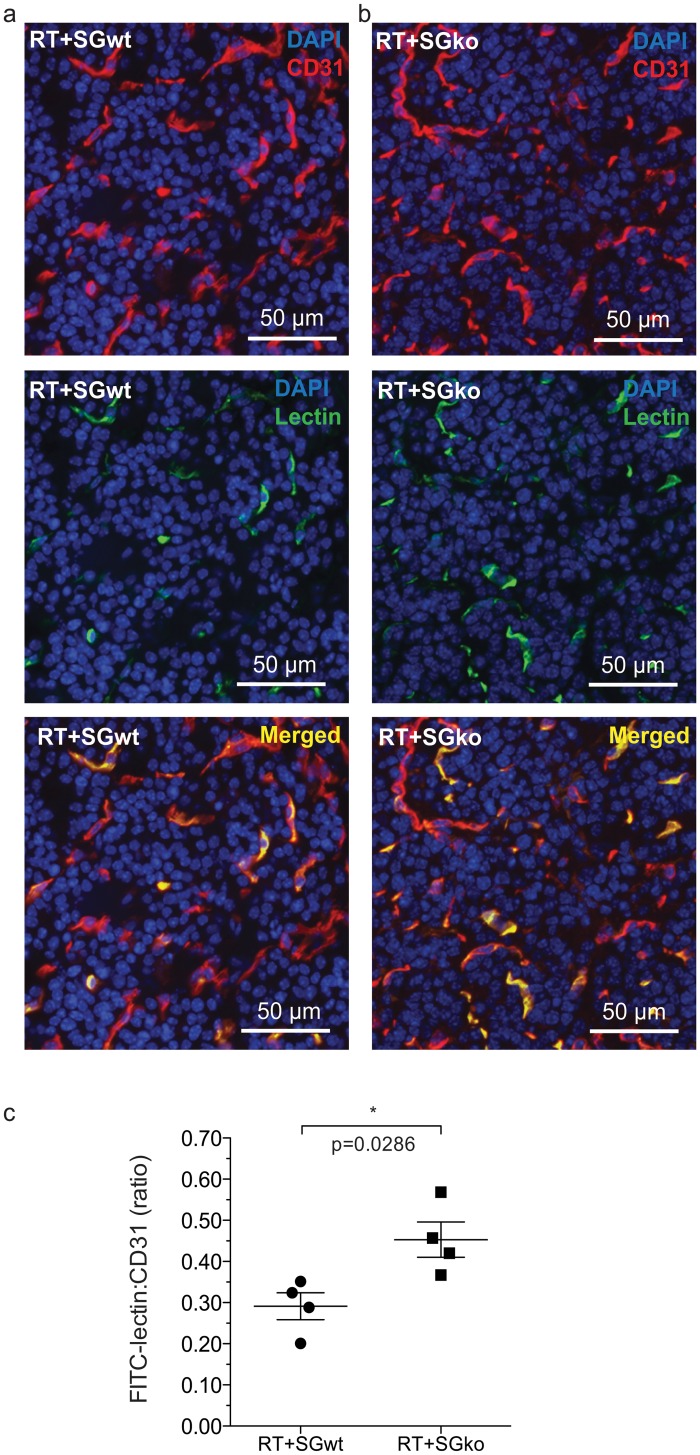
Tumor vessel perfusion. The vascular function is increased in tumors of RT^pos^SG^ko^ versus RT^pos^SG^wt^ mice. 15 week RT^pos^SG^wt^ (a) and RT^pos^SG^ko^ (b) mice were perfused with FITC-lectin, which was introduced to the circulation prior to sacrifice of the animals and excision of the pancreas. Tumor sections were stained for the endothelial marker CD31 (red), the localization of which was compared to lectin (green) that had immobilized on the luminal side of endothelial cells in perfused vessels. The ratio between immobilized lectin and CD31 staining was calculated to determine the fraction of perfused, and thus functional vessels (c). Each data point in represents an individual animal. Statistical analysis was performed using a two-tailed Mann-Whitney test. Error bars represent mean ± SEM.

To assess whether lack of serglycin has any additional effect on the tumor vasculature we initially measured vessel density by staining for the endothelial marker CD31. We observed no difference in the CD31 positive area indicating the same amount of vessels in RT2^pos^SG^ko^ and RT2^pos^SG^wt^ mice (S2 Fig). In order to obtain information of the vascular function, we performed *in vivo* perfusion on tumor bearing mice at 15 weeks with FITC-coupled lectin and subsequently counterstained sectioned tumor tissue for the endothelial marker CD31 (Fig 3a and 3b). Whilst all endothelial cell structures are CD31 positive, the FITC-lectin staining can just be traced to areas that are perfused, indicating that the blood vessels are luminized and are without the obstructions or dead ends that are caused by the dysregulated formation of the vasculature in a highly pro-angiogenic microenvironment. The FITC-lectin stained area was larger in RT2^pos^SG^ko^ (Fig 3b) than in RT2^pos^SG^wt^ mice (Fig 3a), and by comparison of the FITC-lectin and CD31 positive areas we calculated a higher FITC-Lectin:CD31 ratio in the RT2^pos^SG^ko^ than that of RT2^pos^SG^wt^ mice (Fig 3c). The higher ratio seen in RT2^pos^SG^ko^ tumors showed that a greater percentage of the blood vessels were perfused, which is an indication of better blood vessel functionality.

### Levels of VEGF and HGF are reduced in serglycin deficient RIP1-Tag2 mice

**Fig 4:**
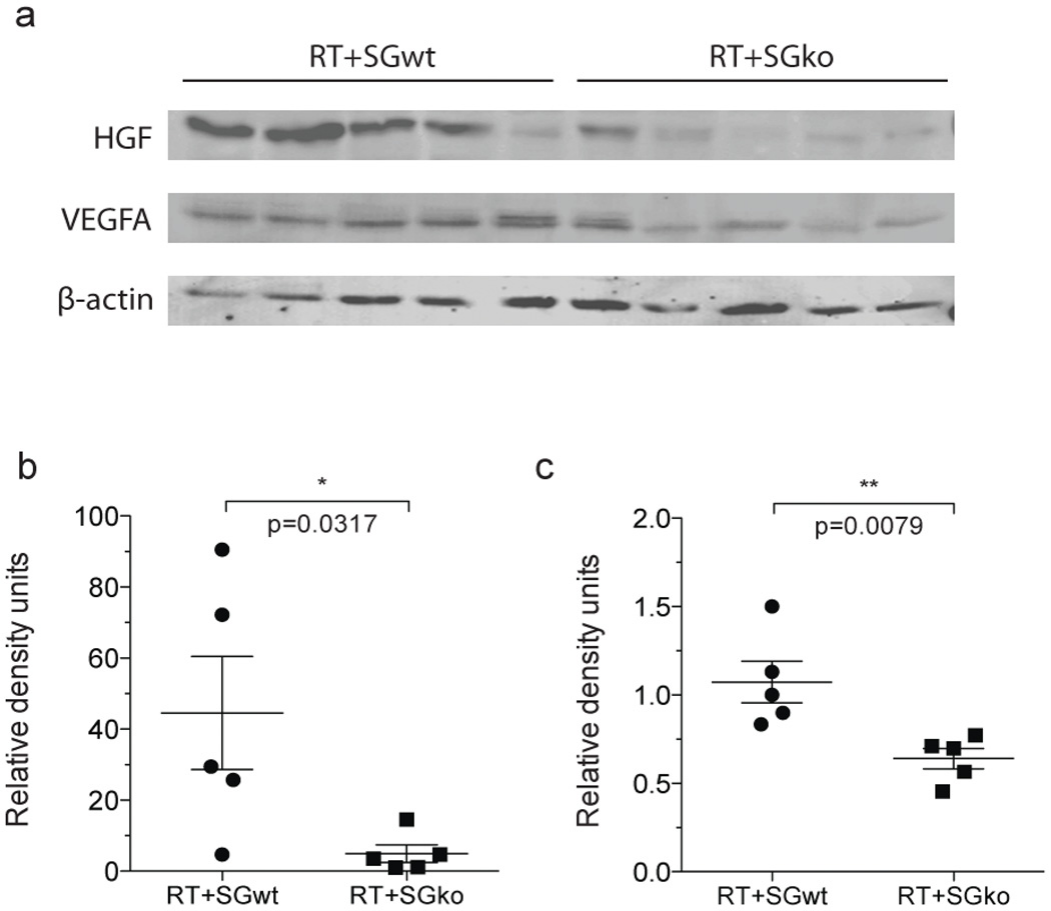
Protein levels of HFG and VEGF in tumor tissue. The levels of HGF and VEGF are significantly decreased in the tumors of serglycin deficient RIP1-Tag2 mice. Lysed tumor material from 15w RT^pos^SG^wt^ and RT^pos^SG^ko^ mice was probed for HGF and VEGF by western blotting (a). HGF (b) and VEGF (c) were significantly less abundant in the serglycin deficient tumors. Values were normalized to beta-actin (a). Each data point in represents an individual animal. Statistical analysis was performed using a two-tailed Mann-Whitney test. Error bars represent mean ± SEM.

The increased vessel function and the decrease in the number of angiogenic islets that we observed suggest a more balanced angiogenic environment in a serglycin deficient tumor environment. To test this, we measured the levels of the two potent pro-angiogenic factors HGF and VEGF. HGF has previously ben shown to be present in lower amounts in tumors with a low serglycin expression [16] and VEGF is the most studied pro-angiogenic factor. We performed western blotting on lysates from whole tumors in RT2^pos^SG^wt^ and RT2^pos^SG^ko^ mice. In RT2^pos^SG^ko^ mice and observed a significant decrease in the levels of both HGF (Fig 4a and 4b), and VEGF (Fig 4a and 4c). This supports the hypothesis of a less pro-angiogenic environment in serglycin deficient tumors.

### Serglycin is highly expressed in RIP1-Tag2 tumors

**Fig 5:**
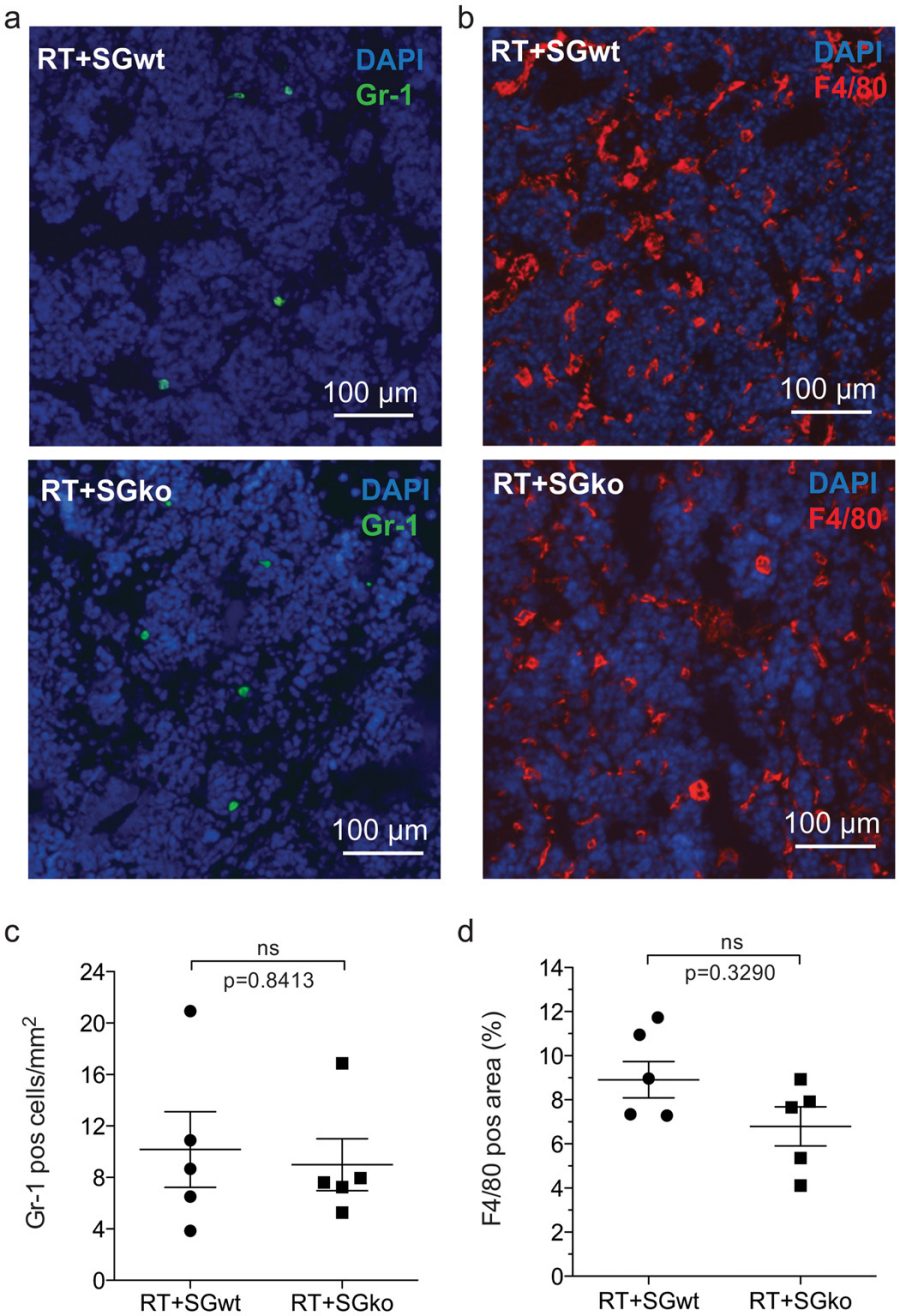
Presence of inflammatory cells in tumor tissue. Macrophage and neutrophil infiltration are unaffected by loss of serglycin in RIP1-Tag2 tumors. Tumor sections from 15w RT^pos^SG^wt^ and RT^pos^SG^ko^ mice were immunostained from the neutrophil and macrophage markers Gr-1 (a) and F4/80 (b) respectively. For Gr-1, the number of positive cells/mm^2^ was calculated (c) and there was no difference in infiltration of neutrophils between the two groups. For F4/80, the area of positive staining was measured (d) and although there was a slight trend to decreased macrophage infiltration in serglycin deficient animals, this was not significant. Each data point in represents an individual animal. Statistical analysis was performed using a two-tailed Mann-Whitney test. Error bars represent mean ± SEM.

Since there is currently no antibody available against serglycin that gives reproducible results in our model, we chose to address expression of serglycin by performing qPCR on material from whole tumor lysates of RT^pos^SG^wt^ and RT^pos^SG^ko^ mice. Spleen was used as a positive control as it is known to express high levels of serglycin [19]. We observed a high expression of serglycin in tumor tissue of serglycin wild type mice (S3 Fig), while no signal was detected in serglycin knockout mice (undetectable Ct value). To determine whether expression of serglycin was from tumor cells, we performed qPCR on cultured beta-TC-6 cells derived from a RIP-Tag mouse inulinoma. Relative to whole tumor tissue, expression of serglycin by the tumor cells was 570x lower (S3 Fig), which suggests that the major contribution of serglycin in the tumor tissue is likely to come from the stroma. We therefore analyzed macrophages and neutrophils, which are two of the inflammatory cells present in vast numbers in the tumor stroma and are thought of as pro-tumorigenic. To study the presence of macrophages and neutrophils in the tissue, tumor sections from RT2^pos^SG^wt^ and RT2^pos^SG^ko^ mice were immunostained against the neutrophil marker Gr-1 (Fig 5a) and the macrophage marker F4/80 (Fig 5b). We did not find any difference in the infiltration of either of the cell types between the genotypes (Fig 5c and 5d). These results indicate that loss of serglycin has no profound effect on the recruitment process of these two major inflammatory cell types, however this does not address any functional differences.

## Discussion

This is the first study where the role of serglycin has been examined in an *in vivo* model of spontaneous tumor development. To address how loss of serglycin affects tumor progression we used the well-characterized RIP1-Tag2 mouse model [17]. We assessed total tumor burden, proliferation and apoptosis, and observed that tumors grew larger in the RT2^pos^SG^ko^ than tumors in the RT2^pos^SG^wt^ group, an effect mainly caused by an enhanced proliferative activity and not by less apoptosis in the tumors. Loss of serglycin resulted in a lower number of angiogenic islets, an enhanced perfusion of the tumor vasculature and decreased levels of VEGF and HGF, suggesting that the presence of serglycin influences angiogenesis and vascular function.

The effect of serglycin expression on tumor growth and tumor cell proliferation has been addressed in several different *in vivo* and *in vitro* models to date. In their study of HCC patient material, He *et al*. [3] saw no correlation between serglycin expression levels and the tumor number or tumor size. Purushothaman and Toole [16] on the other hand observed that the xenografted tumors formed by a multiple myeloma cell line where serglycin had been knocked-down grew smaller than those expressing high levels of serglycin, although proliferation of these cells was unaffected by serglycin expression *in vitro*. A similar observation has also been made in NPC [5] where serglycin expression had no effect on proliferation, however overexpression of serglycin in a breast cancer cell line resulted in a modest increase in proliferation *in vitro* after an extended growth period [4]. In our study using RIP1-Tag2 mice, we in contrast observed larger tumors and increased proliferation upon loss of serglycin. As we did not see any difference in frequency of apoptotic cells between the genotypes we conclude that the enhanced tumor volume in serglycin deficient mice was due to increased proliferation. Previous studies investigating the role of serglycin in tumor progression have focused on the expression of serglycin solely by the tumor cells. We however have used a model that is completely serglycin deficient that takes into account the contribution of serglycin from the entire tumor environment. While we observed a modest expression of serglycin by beta-TC-6 insulinoma cells *in vitro*, very high expression was seen in material isolated from whole tumor tissue, which was comparable to the high levels observed in spleen. This suggests that the major contributors of serglycin in the lesion are not the tumor cells *per se*, but rather are cells within the tumor stroma. This, together with the diverse models used, may account for the differences seen between our investigation and previous studies of the role of serglycin in tumorigenesis.

Tumors are rich in vasculature, however due to the constantly excessive amounts of pro-angiogenic factors, angiogenesis is highly active and the formed tumor vasculature is distorted and has a poor function [15]. As vessels with a better function more efficiently supply the tissue with blood, the elevated proliferation we observed in RT^pos^SG^ko^ mice may be facilitated by an increased availability of nutrients and oxygen. To examine this, we measured tumor vessel density and vessel function. While we did not see any difference in CD31 density, we observed enhanced tumor vascular perfusion and a decreased number of angiogenic islets in serglycin deficient mice, which suggest a less pro-angiogenic environment. In a previous study into the role of angiopoietin-1 and -2 in the RIP1-Tag2 model [20], increased tumor vascular perfusion was positively correlated to an increase in proliferation of tumor cells and an enlarged tumor volume. Consistent with this, in our investigation we saw the same link between an increase in tumor vessel perfusion, and increased volume and proliferation in tumors of RT2^pos^SG^ko^ mice. A role for serglycin in regulating tumor vasculature has previously been suggested by studies of HCC patient material [3] and in an *in vivo* xenograft model of multiple myeloma [16]. In both studies, tumors with low serglycin expression were less vascularized than those with high serglycin expression which points to a less pro-angiogenic environment where serglycin levels are low. Our results showing a better functionality of the tumor vasculature in the RT2^pos^SG^ko^ group go well in line with the results from these studies since a consequence of a lower angiogenic pressure is the formation of a more mature vasculature with a better function [15].

One of the causes of a reduction in angiogenic pressure can be a decrease in the amount of pro-angiogenic effectors. To test this in our model, we measured the levels of the pro-angiogenic factors HGF and VEGF by western blotting and observed a significant reduction of both. The decreased levels of HGF that we observed in the tumors of serglycin deficient mice are in agreement with the study of multiple myeloma by Purushothaman *et al*. [16]. Our investigation for the first time demonstrates a link between loss of serglycin and reduced levels of VEGF in tumor tissue. A reduction of two such potent pro-angiogenic factors may shift the balance between pro- and anti-angiogenic molecules closer to angiostatic equilibrium, thereby allowing the vessels to mature and form patent vasculature with luminized endothelial structures. The decreased presence of HGF and VEGF could therefore explain the increased vascular function that we observed in RT^pos^SG^ko^ mice.

Macrophages and neutrophils are important as providers of pro-angiogenic mediators to the tumor environment, as both cell types are known to express both VEGF and HGF [21–24] and are present in tumor tissue in large numbers [25]. A lack of serglycin may affect both the recruitment process of these inflammatory cells to the tumor as well as the function of the cells once residing in the tumor tissue. We therefore counted the number of infiltrating macrophages and neutrophils and did not find any differences in the amount of these in the tumors between the two genotypes, which indicates that the recruitment of these cell types is unaffected by loss of serglycin. This does not however rule out any functional differences that may contribute to tumor development such as the storage and release of pro-angiogenic mediators including VEGF and HGF, which warrants further investigation.

In the present study, we have addressed how serglycin affects spontaneous tumor progression in an immunocompetent mouse model. We have analyzed late stage tumors in wild type and serglycin deficient mice in the RIP1-Tag2 model at a stage where invasive carcinomas have developed. Our results show that serglycin influences tumor growth, angiogenesis, tumor vascular functionality and the availability of the pro-angiogenic modulators VEGF and HGF. Our data for the first time indicate that serglycin may act as a factor that can suppress spontaneous tumor expansion and highlight its involvement in tumor angiogenesis. Our study further strengthens serglycin as a relevant molecule for more extensive mechanistic studies in the context of cancer pathology.

## Supporting Information

S1 Fig Total frequency of lesions in pancreatic tissue. Tumors and angiogenic islets were dissected from whole pancreas and the frequency of both types of lesion was measured. The number of tumors and angiogenic islets were summed for RT^pos^SG^wt^ and RT^pos^SG^ko^ and a significant difference in the total number of lesions between the groups was observed (a). Lesions grouped into number of angiogenic islets and number of tumors (b). Statistical analysis was performed using a two-tailed Mann-Whitney test. Error bars represent mean ± SEM.
(EPS)

S2 Fig Quantification of the endothelium. Tumor tissue was stained for the endothelial marker CD31 in both genotypes and the positive area was measured. There was no significant difference between the genotypes. Statistical analysis was performed using a two-tailed Mann-Whitney test. Error bars represent mean ± SEM.
(EPS)

S3 Fig Relative expression of serglycin transcript. The expression of serglycin (Srgn) in whole tumor was determined relative to serglycin levels in cultured beta-TC-6 cells (a). Arbp was used as a housekeeping gene and spleen was used as a positive control. The relative expression in whole tumor was 571x greater than in beta-TC-6 cells (a). CT, ΔCT and ΔΔCT values are shown in the table (b).
(EPS)
